# Incidence and predictors for chemotherapy modifications and their impact on the outcome of ovarian cancer patients

**DOI:** 10.1007/s00404-022-06813-9

**Published:** 2022-11-03

**Authors:** Sandra Hatsy, Christine Brambs, Marion Kiechle

**Affiliations:** Department of Obstetrics and Gynaecology, Technical University Munich, Munich, Germany

**Keywords:** Ovarian cancer, Chemotherapy, Modification, Delay

## Abstract

**Purpose:**

Chemotherapy (CTX) is an important part of the treatment strategy of stage II–IV ovarian cancer. CTX modifications, such as delays, dose reductions or premature terminations might have a negative impact on overall survival (OS) and progression free survival (PFS). The goal of this study was to determine the incidence and predictors of CTX modifications and their influence on survival.

**Methods:**

An observational retrospective cohort analysis of 192 ovarian cancer patients who were treated at the Department of Obstetrics and Gynaecology, Technical University Munich, Germany, according to international guidelines was performed including from 2009 to 2013. A potential association between patient and disease characteristics and CTX modifications was tested with multivariate logistic regression. OS and PFS were estimated by Kaplan–Meier analysis.

**Results:**

44.8% (86/192) received a modification of CTX. 34 (17.7%) women discontinued CTX prematurely, 17 (8.9%) underwent a dose reduction, 16 (8.3%) experienced a CTX delay and 10 (5.2%) had both a delay and a dose modification. In nine (4.7%) patients, the dose needed to be divided. Leukopenia (*p* < 0.001) and anaemia (*p* = 0.003) were significantly more common in patients with CTX modifications. Significant predictors for CTX modifications were a history of thrombosis or embolism (*p* < 0.001) and residual tumour postoperatively (*p* = 0.003).

Patients with CTX modifications showed a significantly lower OS as well as PFS (*p* < 0.001), even after adjustment for prognostic factors such as age, body-mass-index, residual tumour, histology, FIGO stage and grading (*p* = 0.005 for OS and *p* = 0.001 for PFS).

**Conclusion:**

CTX modifications have a negative impact on survival. Significant predictors for such modifications are a history of thrombosis or embolism and the presence of residual postoperative tumour. Further studies are needed to avoid CTX modifications and to improve survival of ovarian cancer patients.

## What does this study add to the clinical work

**Table:** 

Knowledge about the prognostic significance of chemotherapy modifications enables physicians to educate patients about the importance of guideline-based therapy, which could improve compliance. Identifying the predictors of chemotherapy modifications allows physicians to intervene at an early stage and establish efficient side-effect management to reduce the need of chemotherapy alterations.	

## Introduction

Ovarian cancer is the seventh most common cancer in women worldwide [1]. In Germany, it is the second most frequent malignant genital tumour after endometrial carcinoma, accounting for 3.3% of all malignant neoplasms in women. Due to the mostly unspecific and subtle symptoms particularly in early disease, ovarian cancer is commonly diagnosed in advanced stages. The established treatment regimen consists of a primary surgery with the goal of a complete removal of all macroscopically visible tumour followed by six adjuvant cycles of CTX consisting of carboplatin AUC (area under the curve) 5 and paclitaxel 175 mg/m^2^ for 6 cycles every 3 weeks. Substances such as topotecan, gemcitabine or pegylated liposomal doxorubicin are used in the treatment of a recurrence [2]. Due to the potential side effects of CTX, modifications of the standard CTX regimen may be necessary [3–8]. The evidence regarding the effects of such modifications on patient survival is controversial.

In the course of this study, the incidence of CTX modifications, their predictive factors and their potential influence on the OS and PFS in ovarian cancer patients were analysed. Furthermore, prognostic factors for such modifications were identified and discussed with existing evidence.

## Methods

This study included all patients with a diagnosis of ovarian cancer who had undergone an initial debulking surgery and adjuvant CTX at the Department of Obstetrics and Gynaecology, Technical University Munich, Germany, from 1/2009 to 12/2013. Patients undergoing only adjuvant or additional neoadjuvant CTX were included, as were patients with recurrent disease who underwent chemotherapy (first and further recurrences). Exclusion criteria were patients with borderline tumours and non-epithelial malignancies, benign neoplasms, patients who did not receive CTX for various reasons (refused therapy, death prior to chemotherapy), patients who underwent neoadjuvant CTX only and patients with insufficient documentation or loss of information. All data were retrospectively extracted from the medical records (clinical databases, reports of the Bavarian tumour register and the interdisciplinary gynaecological tumour board).

The following epidemiological and clinical parameters were collected: date of birth, age, height, weight and body-mass index (BMI) at the time of diagnosis, pre-existing diseases (heart diseases, vascular diseases, respiratory diseases, thyroid diseases, diabetes mellitus, gastrointestinal diseases, mental disorders, neuropathy, gynaecological diseases including breast cancer), the perioperative risk according ASA-classification (American Society of Anaesthesiologists), date of recurrence or progression of disease diagnosed on the basis of radiological and/or laboratory results, date of death or date of last follow-up. Tumour-specific data included the stage of disease (FIGO), histology, grading and the existence of residual tumour after surgery (R1/R2 or R0). Details regarding the CTX were a neoadjuvant versus adjuvant administration, the chemotherapeutical agents used, the number of cycles administered and the side effects of all grades such as gastrointestinal side effects, polyneuropathy, haematological side effects, fever or infections, pain, skin und mucosal irritations, lymphocele or lymphoedema, sleeping disorders, fatigue, shortness of breath and allergic reactions.

The following CTX modifications were identified: premature termination of CTX, delays in the administration of CTX (by at least > 24 h), dose reductions, both delay as well as dose reductions, dose splitting (e.g. administration of 50% of the dose in shorter intervals).

### Statistical analysis

In course of the study, a retrospective analysis on patients who received first-line CTX and in case of recurrent disease, second or further line CTX was performed. All analyses were performed using the software “SPSS Statistics 24” (Statistical Package for the Social Sciences) from IBM (International Business Machines Corporation, Armonk, NY, USA).

The Kolmogorov–Smirnov test was used to test the normal distribution on quantitative parameters, Wilcoxon-Mann–Whitney test (for quantitative and ordinally scaled variables) or *χ*^2^ test (for nominally scaled parameters) was performed to compare different patient groups. The Kruskal-Wallis test compared independent groups according to quantitative and qualitative characteristics. Survival analyses were performed and presented using the Kaplan–Meier method, the significance level was confirmed with the Log-Rank test. Survival times are indicated as median and average survival with 95% confidence intervals and estimated 5-year survival. OS was defined as the period from the date of the initial surgery to the date of death; PFS was defined as the period from the date of the initial surgery to a confirmed recurrence or progression of disease. Women still alive or without any disease progression were censored at the last follow-up.

The influence of prognostic variables on survival was analysed using the Cox-regression model. Results are indicated as HR (hazard ratio) with 95% confidence intervals. The level of significance for all statistical tests was < 0.05.

### Data interpretation

Several studies were identified through systematic research on publication platforms (PubMed, Google Scholar) and used for comparison to results of this study.

## Results

Of 252 primarily documented patients with an ovarian tumour who underwent surgery at the Technical University Munich from 2009 to 2013, 192 were eligible and included in statistical analyses. 60 patients were excluded for several reasons (malignancies of non-epithelial histology, benign neoplasms, patients with neoadjuvant CTX only, patients without CTX, patients with insufficient documentation). The median age at the time of diagnosis was 64 years (range 31–86). The median body-mass index was 23.84 kg/m^2^ (range 14.8–45.5). The majority of patients (*n* = 173, 90.1%) was diagnosed with advanced-stage disease (FIGO IIb-IV), most of them at FIGO stage IIIc (*n* = 115, 59.9%) and IV (*n* = 39, 20.3%). Most patients presented with a serous histology “high grade” (*n* = 143, 74.5%), some were serous “low grade” (*n* = 4, 2.1%) followed by endometrioid (*n* = 15, 7.8%) and mixed entities (*n* = 10, 5.2%). The other histological types included were clear cell (*n* = 8, 4.2%), mucinous (*n* = 5, 2.6%) or tubular (*n* = 1, 0.5%). Six neoplasms (3.1%) could not be assigned to a histological entity (“others”). Most patients (*n* = 159, 82.8%) had poorly differentiated neoplasms (G3), 13.5% (*n* = 26) had moderately differentiated tumours (G2). The remaining tumours were well-differentiated (G1, *n* = 6, 3.1%) or undifferentiated (G4, *n* = 1, 0.5%). 103 patients (53.6%) did not have any residual disease after surgery (R0), in 89 women (46.4%) a microscopic or macroscopic tumour residual disease remained postoperatively (R1 or R2, Table 1). 174 patients (90.6%) were treated with adjuvant CTX, 18 women (9.4%) underwent neoadjuvant as well as adjuvant chemotherapeutical treatment.

**Table 1:** Patient characteristics (*n* = 192)

	*n*	Percentage(%)
FIGO		
I	1	0.5
Ia	6	3.1
Ib	0	0
Ic	7	3.6
II	1	0.5
IIa	4	2.1
IIb	4	2.1
IIc	2	1
III	1	0.5
IIIa	6	3.1
IIIb	6	3.1
IIIc	115	59.9
IV	39	20.3
Histology		
Serous (high grade)	143	74.5
Serous (low grade)	4	2.1
Endometrioid	15	7.8
Clear cell	8	4.2
Mucinous	5	2.6
Tubular	1	0.5
Mixed entity	10	5.2
Others	6	3.1
Grading		
G1	6	3.1
G2	26	13.5
G3	159	82.8
G4	1	0.5
Residual disease		
R1/R2	89	46.4
R0	103	53.6

The treatment regimen in early stages primarily included chemotherapy with platinum only for six cycles; in advanced stages, patients received six cycles of platinum- and taxane-based CTX. In addition, 78 women (40.6%) received the bevacizumab in addition to the CTX and as a maintenance therapy after the completion of CTX. 1 in 12 patients (8.3%) with a history of thrombosis or embolism received bevacizumab. In case of recurrent disease, additional agents such as topotecan, gemcitabine or pegylated liposomal doxorubicin were administered.

All patients initially received the chemotherapy according to the recommendations of the interdisciplinary tumour board. During the course of treatment, 106 women (55.2%) received CTX recommended in the interdisciplinary tumour board without alterations. 86 patients (44.8%) underwent CTX modifications: 34 women (17.7%) discontinued the CTX prematurely, 17 (8.9%) experienced dose reductions, 16 (8.3%) had cycle delays (> 24 h from original schedule), 10 (5.2%) experienced both dose reductions and delays, and 9 (4.7%) received split (halved dose) CTX.

In 60 patients (31.3%), CTX modifications were implemented during first-line treatment.

Most of the chemotherapeutical side effects were nausea and/or vomiting (13.7%), followed by fatigue (12.3%), polyneuropathy (9.7%), constipation (8.4%), pain (8.2%), leukopenia (8.4%), anaemia (8.4%), irritations of nails/skin/mucosa (5.6%) and fever/infections (5.3%). Other symptoms (around 25%) were diarrhoea, lymphocele/ lymphoedema, shortness of breath, thrombocytopenia, hot flushes, sleeping disorders, hand-foot-mouth syndrome and allergies.

### No modification versus modification

Patients with and without CTX modifications differed significantly in regards to age and residual disease status. Patients with CTX modifications were significantly older than women without any therapy adjustments (*p* = 0.034; median 66 versus 62 years, respectively).

The number of R1 resections was significantly higher in women with CTX modifications. 58.1% (*n* = 50) of patients with therapy adjustments had residual disease after surgery whereas 36.8% (*n* = 39) of patients without CTX modifications had residual disease postoperatively (R1/R2; *p* = 0.003).

The two patient groups displayed the following differences in the past medical history: vascular disease (hypertension, hypercholesterolemia, venous insufficiency, thrombosis/embolism, atherosclerosis) was more common in women who underwent CTX modifications compared to patients without any therapy variations (*n* = 49, 57.0% versus *n* = 44, 41.5%; *p* = 0.003). A history of thrombosis or embolism was present in 12.8% (*n* = 11) of the patients with modifications compared to 0.9% (*n* = 1) in those without modifications (*p* = 0.001).

Women with adjustments in their chemotherapeutical treatment also had significantly more pre-existing psychological conditions (depressions, anxiety disorders) compared to patients without modifications (*n* = 12, 14.0% versus *n* = 5, 4.7%; *p* = 0.025).

Patients with adjustments in their CTX experienced significantly more side effects than patients without CTX modifications (on average 5.4 side effects versus 4.2, respectively; *p* = 0.003). Gastrointestinal symptoms (*n* = 70, 85.4% versus *n* = 65, 67.7%, *p* = 0.006), especially nausea and vomiting (*n* = 62, 75.6% versus *n* = 54, 56.3%, *p* = 0.007), as well as haematological side effects (*n* = 60, 73.2% versus *n* = 30, 31.1%, *p* < 0.001), especially leukopenia (*n* = 39, 47.6% versus *n* = 16, 16.7%, *p* < 0.001) and anaemia (*n* = 34, 41.5% versus *n* = 20, 20.8%, *p* = 0.003) were significantly more common in patients with CTX modifications. Fever or infections were also more common in patients with chemotherapeutical treatment variations (*n* = 27, 32.9% versus *n* = 18, 18.8%, *p* = 0.030).

### Predictors for CTX modifications

Several statistically significant predictors for CTX modifications were identified in the current study: postoperative residual disease, a history of vascular disease (especially thrombosis/embolism), side effects of CTX like gastrointestinal symptoms (especially nausea/vomiting), haematological side effects (in particular leukopenia and anaemia) and fever or infections (Table 2).

**Table 2:** Predictors for chemotherapy modifications

	OR	95% CI	*P* value
Residual tumor mass	2.39	1.33–4.27	0.003
Medical history			
Vascular diseases^a^	1.87	1.05–3.32	0.033
Thrombosis/Embolism	15.4	1.95–121.85	0.001
Mental disorders^b^	2.19	0.94–5.12	0.025
Side effects of chemotherapy			
Gastrointestinal side effects^c^	2.78	1.32–5.87	0.006
Nausea/Vomiting	2.41	1.26–4.60	0.007
Haematological side effects^d^	6	3.12–11.51	< 0.001
Leukopenia	4.54	2.28–9.04	< 0.001
Anaemia	2.69	1.39–5.21	0.003
Fever/Infections	2.13	1.07–4.24	0.03

*OR* odds ratio, 95% *CI* 95% confidence interval

^a^Including hypertension, hypercholesterolaemia, venous insufficiency, thrombosis/embolism, atherosclerosis

^b^Including depression, anxiety disorder

^c^Including nausea/vomiting, constipation, diarrhoea

^d^Including leukopenia, anaemia, thrombocytopenia

### Survival

The median OS of all patients included in this study was 50 months (95% CI 43.1–56.9 months), the average survival was 50.9 months (95% CI 46.4–55.3 months). The median PFS was 27 months (95% CI 22.7–31.3 months), the average PFS was 30.6 months (95% CI 27.2–34 months).

The Kaplan–Meier survival analyses showed a significantly lower OS as well as PFS for patients who underwent CTX modifications compared to patients without any adjustments of the initial CTX regimen (average OS: 59.5 versus 39.8 months, *p* < 0.001; average PFS: 40.5 versus 21 months, *p* < 0.001). The survival analyses did not show any significant differences in OS as well as PFS between the five patient groups in regards to their CTX modifications (premature termination, dose reduction, delay, dose reduction and delay, split dose) (Table 3).

**Table 3:** Kaplan–Meier survival analyses for patients with or without chemotherapy modifications and the five patient groups according their CTX modifications: premature termination, delay, dose reduction, dose reduction and delay, split dose

OS (months)						
	*n*	Mdn. OS	95% CI	Avg. OS	95% CI	5-year-OS
No CTX modification	106			59.5	53.9–65.1	60.2%
CTX modification	86	40	30.6–49.3	39.8	33.8–45.7	20.9%
Sig.	*p* < 0.001					
Termination	34	30	22.6–37.5	33.5	25.1–41.9	9.1%
Delay	16	39	12.4–65.7	44.9	32–57.9	37.5%
Dose reduction	17	40	29.2–50.8	42.1	29.9–54.3	34.8%
Delay + dose reduction	10	50		40.8	30.7–51	0%
Split dose	9	50	0–126.9	33.4	15.2–51.6	18.5%
Sig.	*p* = 0.639					

PFS (months)						
	*n*	Mdn. PFS	95% CI	Avg. PFS	95% CI	5-year-PFS
No CTX modification	106	27	20.5–33.5	40.5	34.3–46.8	36.8%
CTX modification	86	16	14.2–17.8	21	16.8–25.2	7.1%
Sig.	*p* < 0.001					
Termination	34	14	9.1–18.9	13.9	11.1–16.6	0%
Delay	16	16	8.2–23.8	26.6	16.3–36.9	12.5%
Dose reduction	17	16	11–21	18.9	13.5–24.3	0%
Delay + dose reduction	10	22	13.3–30.7	24	16.3–31.7	0%
Split dose	9	18	9.2–26.8	20.7	7.7–33.8	0%
Sig.	*p* = 0.099					

*OS* overall survival, *PFS* progression free survival, *n* number, *mdn*. median, *avg* average, *CTX* chemotherapy, 95% *CI* 95% confidence interval, *sig.* significance

**Fig. 1:**
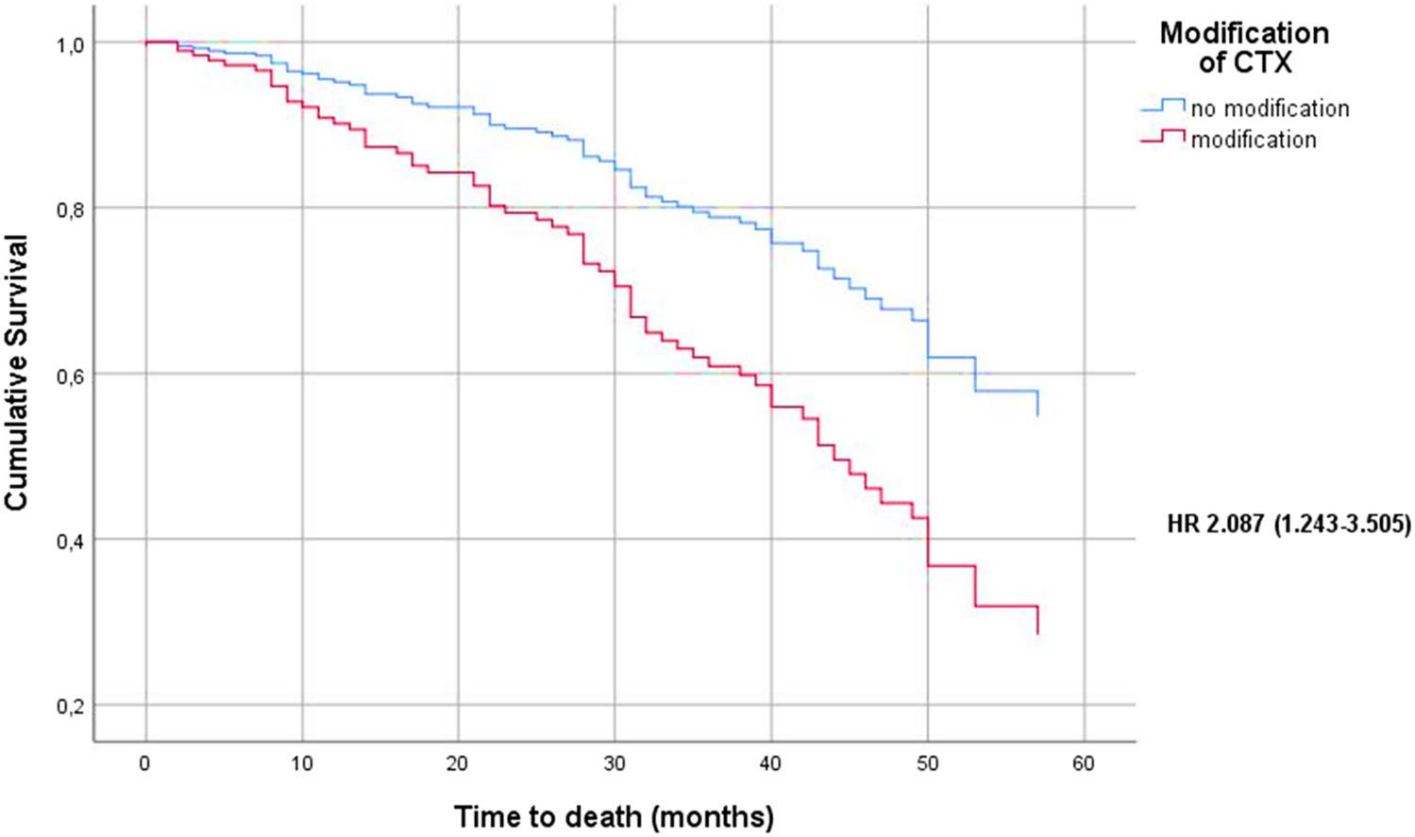
Overall survival (months) of patients with and without chemotherapy modifications after adjustment for risk factors (age, body-mass index at time of diagnosis, residual disease, FIGO stage, histology, grading) using a Cox regression model (*HR* hazard ratio)

**Fig. 2:**
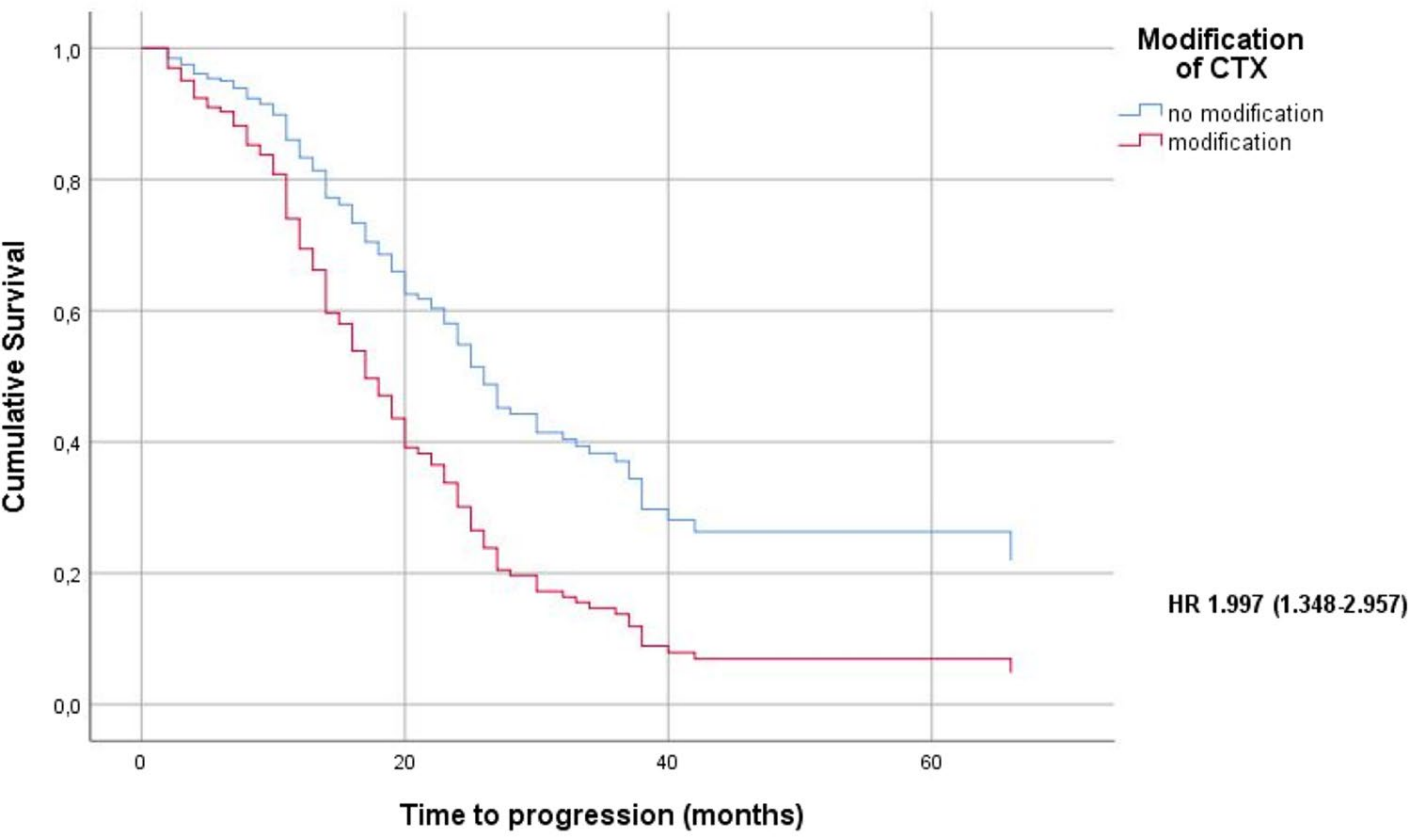
Progression free survival (months) of patients with and without chemotherapy modifications after adjustment for risk factors (age, body-mass index at time of diagnosis, residual disease, FIGO stage, histology, grading) using a Cox regression model (*HR*   hazard ratio)

After adjustment for risk factors (age, body-mass index at time of diagnosis, modification of CTX, residual disease, FIGO stage, histology, grading), the prognostic importance of CTX modifications remained significant in a Cox regression model (Table 4; Figs. 1, 2).

**Table 4:** Prognostic factors on Overall survival and Progression free survival after adjustment for risk factors (modification of CTX, age, body-mass index at time of diagnosis, residual disease, FIGO stage, histology, grading) using a Cox regression model

Prognostic factors	OS		PFS	
	HR. 95% CI	*P* value	HR. 95% CI	*P* value
CTX modification	2.09 (1.24–3.51)	**0.005**	2.00 (1.35–2.96)	**0.001**
Age (years)	1.01 (0.98–1.03)	0.706	1.00 (0.98–1.02)	0.948
Body-Mass-Index (kg/m^2^)	0.99 (0.95–1.04)	0.71	0.97 (0.93–1.00)	0.08
Residual disease	3.06 (1.64–5.74)	** < 0.001**	1.81 (1.19–2.75)	**0.006**
FIGO (I–IIc versus III–IV)	2.76 (0.61–12.46)	0.187	5.71 (2.05–15.85)	**0.001**
Histology		**0.008**		**0.014**
Serous	1		1	
Endometrioid	1.30 (0.37–4.55)	0.681	1.69 (0.70–4.05)	0.241
Clear cell	2.40 (0.64–9.01)	0.196	5.10 (1.66–15.60)	**0.004**
Mucinous	11.42 (2.84–45.99)	**0.001**	1.93 (0.60–6.28)	0.273
Mixed entity	0.58 (0.14–2.40)	0.452	2.37 (1.01–5.57)	**0.048**
Grading		0.374		0.804
G1	1		1	
G2	0.81 (0.16–4.04)	0.802	1.27 (0.41–3.94)	0.684
G3	1.62 (0.35–7.52)	0.536	1.39 (0.49–3.93)	0.533

Significance for all statistical tests was < 0.05

*OS* overall survival, *PFS* progression free survival, *CTX* chemotherapy, *HR* hazard Ratio, 95% *CI* 95% confidence interval

## Discussion

This current study shows a negative influence of modifications of the CTX regimen on OS and PFS in patients with ovarian cancer. In the past, several studies have evaluated the prognostic impact of CTX modifications. The significance of cycle delays during CTX has been addressed by the following studies: Liutkauskiene et al. [7] analysed data from 82 women with FIGO stage III tumours who had already completed 6 cycles of a platinum-based CTX. Among all CTX modifications, cycles delays of more than 10 days were associated with an approximately tripled risk of death (HR 3.3, *p* = 0.016). They reported an indication for CTX delays in 14.6% of their patients (*n* = 12).

Joseph et al. [6] found even a single cycle deferral of an undefined extent to result in a significantly shorter median OS compared to women without a delay (2.5 versus 4 years, respectively; *p* < 0.02) among 184 patients with stage II–IV ovarian cancer. Cycle delays occurred in 45% (*n* = 47) of women who completed a platinum-based combination chemotherapy (*n* = 105).

The study by Seebacher et al. [9] included 165 patients with a completed platinum and taxane-containing CTX. In patients with treatment, postponements of at least 9 days over the entire duration of CTX (*n* = 90), both mortality and recurrence risk were twice as high as in patients without postponements (OS: HR 2.6, *p* = 0.008; PFS: HR 2.9, *p* = 0.001).

In contrast, the study by Nagel et al. [10] did not confirm a negative prognostic significance of cycle delays. There were no differences in PFS (*p* = 0.50) and OS (*p* = 0.76) between the patient groups.

The importance of dose modifications of CTX has also been the subject of several studies. Hanna et al. [8] described a significant increase in the mortality among 325 women with advanced tumours when the CTX dose was reduced to < 85% (HR 1.71, *p* = 0.003).

This is in contrast to results presented by Repetto et al. [11], Olawaiye et al. [12] and Nagel et al. [10] who did not reveal a significant association between the CTX dose and survival in patients with ovarian cancer.

The data on the significance of a premature discontinuation of CTX are also heterogenous.

In a study population of 1932 individuals, Wright et al. [13] showed that a treatment duration of less than 3 months (*n* = 714, 37%) compared to a duration of 3–7 months (*n* = 1218, 63%) is associated with a significantly worse OS (*p* < 0.0001) as well as cancer-specific survival (*p* < 0.0001).

Joseph et al. [6] described no significant negative survival effect of any CTX adjustments, even early discontinuations. However, these were patients with colorectal cancer–the results can therefore not be directly compared to this current study.

Overall, the results of our study are consistent with several other studies [6–9, 13]. In conclusion, a potential prognostic disadvantage of CTX modifications must be assumed. Providers should, therefore, try to avoid any deviations from the initial regimen whenever possible.

To achieve this goal, a better knowledge of the predictors for CTX modifications is essential. In our study, a medical history of thromboembolic disorders was found to be significant (OR = 15.4, *p* = 0.001).

To our knowledge, there is no further study to date that assesses the significance of thromboembolism in the medical history. Abu Saadeh et al. [14] address the significance of a thromboembolism perioperatively or during chemotherapy. 344 patients with ovarian cancer were included, with 33 women (9.7%) suffering from deep venous thrombosis or pulmonary embolism either perioperatively or during CTX. These patients showed a significantly reduced OS compared to women without thromboembolism (34.8 vs. 55.8 months, *p* < 0.001).

A comparison to our results is hardly possible because in our study thromboembolism in the medical history regardless of the treatment of ovarian cancer was recorded and analysed. Our study shows that thromboses and embolisms in the medical history of patients with ovarian cancer can be predictors of CTX modifications and as a further consequence be significant for survival. This may be at least partly explained by the fact that patients with a history of thrombosis and embolism did not receive bevacizumab, except in one case.

Haematological side effects of CTX also have a significant predictive value. In our study, a sixfold increase in risk for CTX modifications was calculated for women who showed clinically relevant changes in haematological parameters (OR = 6, *p* < 0.001), especially leukocytopenia (OR = 4.54, *p* < 0.001) and anaemia (OR = 2.69, *p* = 0.003).

Family et al. [15] confirmed the association of anaemia with dose reductions or cycle deferrals in CTX. Among 3955 patients with different cancer diagnoses, moderate (grade 2, haemoglobin < 10 g/dl) or advanced (grade 3–4, haemoglobin 8 g/dl) anaemia significantly increased the risk of dose reductions and cycle deferrals compared to mild or absent anaemia (OR = 1.45 for grade 2 and OR = 2.02 for grades 3–4).

Khan et al. [16] described that among 50 patients with different cancer diagnoses, 40% developed neutropenia in the course of CTX, which necessitated adjustments to the regimen: cycle deferrals of ≥ 7 days were necessary for 30% of the patients, dose reductions of ≥ 15% were carried out for 20% of the patients.

The retrospective study of Eichbaum et al. [17] focused on the prognostic impact of haemoglobin levels before and during carboplatin/taxane-based chemotherapy in patients with primary ovarian cancer. Among 92 patients, haemoglobin levels throughout chemotherapy showed prognostic relevance in terms of PFS (*p* < 0.05). A haemoglobin level of 11.2 g/dL was found to be a prognostically relevant cut-off.

Gerestein et al. [18] confirmed the prognostic significance of anaemia in terms of overall survival. Among 118 women with advanced-stage epithelial ovarian cancer, the preoperative haemoglobin serum concentration (*p* = 0.012), preoperative platelet counts (*p* = 0.031) and residual disease < 1 cm (*p* = 0.028) were predictive for overall survival.

The prognostic significance of chemotherapy-induced neutropenia has not been fully clarified. Kim et al. [19] analysed 130 patients with ovarian cancer who underwent surgery followed by 6 adjuvant cycles of chemotherapy with carboplatin and paclitaxel. Patients with cycle postponements and dose reductions were excluded from the analysis. No significant difference was observed between the patients with and without neutropenia during chemotherapy (PFS: 34 versus 22 months, *p* = 0.26; OS: 67 versus 56 months, *p* = 0.59).

Tewari et al. [20] even described a survival advantage for patients with chemotherapy-induced neutropenia among 3447 patients with advanced ovarian or primary peritoneal cancer. Neutropenic patients (*n* = 3196) experienced significantly improved survival compared to non-neutropenic patients (*n* = 251). The risk of death decreased by 14% (HR 0.86, *p* = 0.041). Perhaps this effect is an indicator for a good efficacy of the chemotherapy.

Based on our results, we cannot make a statement on the prognostic impact of haematological side effects of chemotherapy.

Patients in our group with postoperative residual disease were twice as likely to undergo CTX modifications than patients with no residual disease (OR = 2.386, *p* = 0.003). To our knowledge, this association has not been found in any other study so far. One potential explanation is that women with residual disease usually suffer from advanced tumours and may display a reduced general condition compared to patients with early-stage carcinomas. CTX modifications may therefore be more common and residual disease might not be an independent predictor of a potential need for CTX modifications. However, the residual disease and the CTX tolerance could also represent independent parameters. Further studies are needed to evaluate this potential association.

There are certain limitations to the current study. The number of patients with CTX modifications (*n* = 86) is small. Analyses between the different modification groups may, therefore, be of limited significance. Given the retrospective design of the study, only patients with sufficient documentation could be included, leading to the exclusion of several patients. The epidemiological and clinical parameters were extracted retrospectively from different documentation sources and were thus also dependent on the quality of the documentation.

Furthermore, modifications were not quantified with sufficient precision. For example, patients with any dose reduction were grouped regardless of the extent and frequency of the reductions. The same applies to cycle delays. Due to the small number of patients, however, the formation of subgroups within each modification group was not considered useful.

In our study, CTX modifications showed a negative impact on OS as well as PFS in patients with ovarian cancer. In this context, CTX modification may contribute to the risk of progression and/or death. Many other variables, such as significantly older age or more frequent previous vascular disease in patients with CTX modifications, could also have an impact on the outcome. It is likely that a combination of several factors is relevant. A precise differentiation is not possible with the available data.

In the future, the value of chemotherapy modification will likely be even more difficult to analyse. The increasing role of tumour biology and the increasingly personalized therapy will give chemotherapy less importance in the long term, while e.g. PARP inhibitors are becoming more relevant in BRCA-mutated patients or patients with positive HRD status (homologous recombination deficit).

## Conclusion

Our study shows a negative influence of adjustments of the standard CTX regimen on OS and PFS in patients with ovarian cancer. A comparison of the different modification groups shows no significant difference, so that CTX modifications can be regarded as prognostically unfavourable, but no statement can be made about the significance of the individual adjustments. Further studies are needed to estimate the relevance of the respective modifications. Predictors of CTX modifications include postoperative residual disease, a history of thromboses and embolisms, gastrointestinal and haematological side effects and fever or infections during the course of CTX.

## References

[1] Reid BM, Permuth JB, Sellers TA (2017). Epidemiology of ovarian cancer a review. Cancer Biol Med.

[2] Leitlinienprogramm Onkologie (Deutsche Krebsgesellschaft, Deutsche Krebshilfe, AWMF): S3-Leitlinie Diagnostik, Therapie und Nachsorge maligner. Ovarialtumoren, Langversion 5.0, 2021, AWMF-Registernummer: 032/035OL, https://www.leitlinienprogramm-onkologie.de/leitlinien/ovarialkarzinom/

[3] Heilmann T, Pfisterer J, Hempel AM, Saß S, Hedderich J, Pujade-Lauraine E (2019). Early treatment modifications improve chemotherapy adherence in ovarian cancer patients ≥70 years. Gynecol Oncol.

[4] Salman L, Ben-Haroush A, Raban O, Yeoshoua E, Sabah G, Jakobson-Setton A (2019). Neoadjuvant chemotherapy treatment modifications in ovarian carcinoma: the impact on surgical outcome and progression-free survival. Ame J Clin Oncol.

[5] Denduluri N, Lyman GH, Wang Y, Morrow PK, Barron R, Patt D (2018). Chemotherapy dose intensity and overall survival among patients with advanced breast or ovarian cancer. Clin Breast Cancer.

[6] Joseph N, Clark RM, Dizon DS, Lee MS, Goodman A, Boruta D (2015). Delay in chemotherapy administration impacts survival in elderly patients with epithelial ovarian cancer. Gynecol Oncol.

[7] Liutkauskiene S, Janciauskiene R, Jureniene K, Grizas S, Malonyte R, Juozaityte E (2015). Retrospective analysis of the impact of platinum dose reduction and chemotherapy delays on the outcomes of stage III ovarian cancer patients. BMC Cancer.

[8] Hanna RK, Poniewierski MS, Laskey RA, Lopez MA, Shafer A, van Le L (2013). Predictors of reduced relative dose intensity and its relationship to mortality in women receiving multi-agent chemotherapy for epithelial ovarian cancer. Gynecol Oncol.

[9] Seebacher V, Reinthaller A, Koelbl H, Concin N, Nehoda R, Polterauer S (2017). The impact of the duration of adjuvant chemotherapy on survival in patients with epithelial ovarian cancer - a retrospective study. PLoS ONE.

[10] Nagel CI, Backes FJ, Hade EM, Cohn DE, Eisenhauer EL, O'Malley DM (2012). Effect of chemotherapy delays and dose reductions on progression free and overall survival in the treatment of epithelial ovarian cancer. Gynecol Oncol.

[11] Repetto L, Pace M, Mammoliti S, Bruzzone M, Chiara S, Oliva C (1993). The impact of received dose intensity on the outcome of advanced ovarian cancer. Eur J Cancer.

[12] Olawaiye A, Java J, Krivak T, Bookman M, Herzog T (2012). Does maintaining adjuvant chemotherapy dose intensity have an impact on the outcome of treatment in ovarian cancer patients? A Gynecologic Oncology Group study. Gynecol Oncol.

[13] Wright JD, Doan T, McBride R, Jacobson JS, Hershman Dl (2008). Variability in chemotherapy delivery for elderly women with advanced stage ovarian cancer and its impact on survival. British J Cancer.

[14] Saadeh FA, Norris L, O’Toole S, Gleeson N (2013). Venous thromboembolism in ovarian cancer. Incidence, risk factors and impact on survival. Eur J Obstet Gynecol Reprod Biol.

[15] Family L, Xu L, Xu H, Cannavale K, Sattayapiwat O, Page JH (2016). The effect of chemotherapy-induced anemia on dose reduction and dose delay. Support Care Cancer.

[16] Khan S, Dhadda A, Fyfe D, Sundar S (2008). Impact of neutropenia on delivering planned chemotherapy for solid tumours. Eur J Cancer Care.

[17] Eichbaum MH, Weiss LM, Bruckner T, Schneeweiss A, Sinn HP, Gebauer G, Fersis N, Kussmaul J, Sohn C (2009). Prognostic impact of hemoglobin levels before and during carboplatin/taxane-based chemotherapy in patients with primary invasive epithelial ovarian cancer. Med Sci Monit.

[18] Gerestein CG, Eijkemans MJ, de Jong D, van der Burg ME, Dykgraaf RH, Kooi GS, Baalbergen A, Burger CW, Ansink AC (2009). The prediction of progression-free and overall survival in women with an advanced stage of epithelial ovarian carcinoma. BJOG.

[19] Kim JJ, Park JY, Kim DY, Kim JH, Kim YM, Nam JH, Kim YT (2010). Is chemotherapy-induced neutropenia a prognostic factor in patients with ovarian cancer?. Acta Obstet Gynecol Scand.

[20] Tewari KS, Java JJ, Gatcliffe TA, Bookman MA, Monk BJ (2014). Chemotherapy-induced neutropenia as a biomarker of survival in advanced ovarian carcinoma: an exploratory study of the gynecologic oncology group. Gynecol Oncol.

